# The precision of epidemiological investigation of COVID-19 transmission in Shanghai, China

**DOI:** 10.1186/s40249-021-00849-w

**Published:** 2021-05-05

**Authors:** Ying Shi, Hong-Lin Jiang, Mei-Xia Yang, Lin-Juan Dong, Yue Chen, Yi-Biao Zhou, Qing-Wu Jiang

**Affiliations:** 1 Fudan University School of Public Health, Building 8, 130 Dong’an Road, Shanghai, 200032 China; 2 Key Laboratory of Public Health Safety, Fudan University, Ministry of Education, Building 8, 130 Dong’an Road, Shanghai, 200032 China; 3 Fudan University Center for Tropical Disease Research, Building 8, 130 Dong’an Road, Shanghai, 200032 China; 4 Xuhui Center for Disease Control and Prevention, Shanghai, China; 5 Community Healthcare Center of Bansongyuan Street, Huangpu District, Shanghai, China; 6 School of Epidemiology and Public Health, Faculty of Medicine, University of Ottawa, 451 Smyth Road, Ottawa, ON K1H 8M5 Canada

**Keywords:** COVID-19, Outbreak, Precision of epidemiological investigation, Shanghai

## Abstract

**Background:**

Shanghai had a local outbreak of COVID-19 from January 21 to 24. Timely and precise strategies were taken to prevent further spread of the disease. We discussed and shared the experience of COVID-19 containment in Shanghai.

**Process:**

The first two patients worked at two hospitals but no staff from the two hospitals were infected. The suspected case and his two close contacts were confirmed to be infected within 12 h. The testing rate of individuals was low. The scope of screening was minimized to two related districts and the close contact tracing was completed within 12 h, which were precise and cost-effective.

**Conclusions:**

Active monitoring, precise epidemiological investigation and timely nucleic acid testing help discover new cases, minimize the scope of screening, and interrupt the transmission.

**Graphic abstract:**

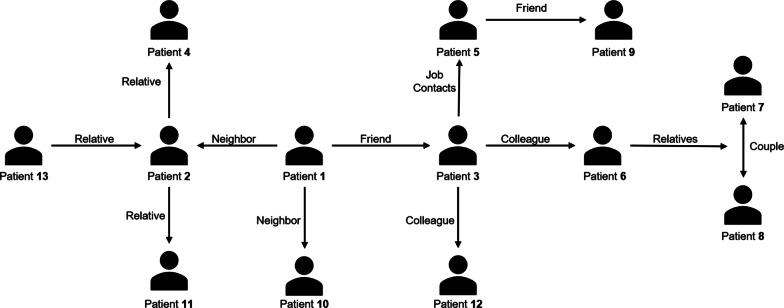

## Background

A local transmission of severe acute respiratory syndrome coronavirus 2 (SARS-CoV-2) occurred in Shanghai in January 2021. Timely and precise prevention and control measures were taken to block the transmission and contain the epidemic. We discussed the strategies and measures of COVID-19 management in Shanghai, and shared the experience for controlling the small-scale outbreaks, especially in metropolis.

## Process of the outbreak in Shanghai

A worker at the Shanghai Cancer Center affiliated with Fudan University had a routine coronavirus test on January 20, 2021, and was suspected of having COVID-19. The health authorities immediately implemented a series of prevention and control measures including detailed epidemiological investigations and centralized quarantine of suspected close contacts. On January 21, the worker was confirmed to be the first new local case (Patient 1) of this outbreak. The second and third infected cases are his neighbor (Patient 2) and friend (Patient 3). Patient 4 is a relative of Patient 2. Patient 5 of the cluster resided in the hotel where Patient 3 worked, and a colleague of Patient 3 was confirmed to be infected with SARS-CoV-2 (Patients 6). They were all diagnosed on January 21, and connected to the subsequent patients.

**Fig. 1:**
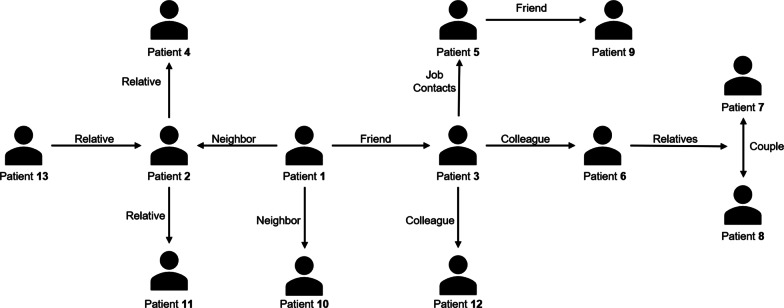
The relationships among new local COVID-19 cases in Shanghai outbreak

Three newly infected cases were reported on January 22, relatives (Patients 7 and 8) of Patient 6 and a friend (Patient 9) of Patient 5. Three more cases occurred on January 23. Patient 10 lives in the same community with Patient 1 and was suspected to have a contact with Patient 1. Patient 11 is a relative of Patient 2. Patient 13 was reported on January 24, and is a relative of Patient 2 as well. A total of 13 confirmed COVID-19 were all linked as shown in Fig. 1. The outbreak was controlled without a widespread transmission.

## Precision of epidemiological investigation

The results of epidemiological investigation show that Patient 1 is a male and 56 years old, and he is a support crew in the Shanghai Cancer Center affiliated with Fudan University and lives in Huangpu Distinct, Shanghai. Patient 2 is a male and 53 years old, and he is a staff in Renji Hospital affiliated with Shanghai Jiaotong University, school of medicine. He lives in the same neighborhood with patient 1. Patient 3 is a female and 48 years old, and she is a friend of Patient 1. The epidemiological investigation also shows that the close-contact mode might be the route of transmission of this outbreak in Shanghai.

All the cases were in the early stage of COVID-19 infection. After diagnosed, they were transferred to Shanghai Public Health Clinical Center for further treatment, then had some symptoms such as fever, cough or expectoration. There are 12 cases with ordinary type and 1 case with light type, none cases with intensive or critical type. Three cases were more than 70 years old, and two of whom were 84 years old. They are all alive and some of cases had hypertension, diabetes or fat.

Shanghai acted quickly in response to this round of COVID-19 outbreak. Epidemiological investigation and aggressive contact tracing were instantly initiated when a suspected case with COVID-19 infection was found on January 20 at 3:00 pm. The suspected case and his two close contacts were confirmed to be infected within 12 h. Usually, the overall test turnaround often exceeds 48 h for transporting samples to the laboratory and returning results to the originator [1]. All staff from the Shanghai Cancer Center Affiliated with Fudan University, where Patient 1 works, and the Renji Hospital, where Patent 2 works, were tested negative for SARS-CoV-2 during two rounds of screening [2]. The first round was conducted on January 21, and the second one on January 23. It indicates that strict protection measures are crucial in preventing healthcare workers from infection and containing the transmission. Other cases were all related to patients diagnosed on January 21 and they are friends, neighbors, relatives or coworkers, suggesting that this local transmission occurred mainly through close daily contacts. Reducing gatherings, wearing masks, and maintaining hand hygiene are the most important intervention measures [3].

As of January 24, 37 592 individuals had been tested, only accounting for 5.78% of about total 650 000 population of Huangpu District (reported in 2018), Shanghai. Only close contacts and residents in the same or surrounding communities and work places with infected cases were tested for the virus. The testing rate was lower as compared with other cities in China. While in another city, a total of 10.9 million people was screened, and the testing rate is nearly 100% [4]. It is necessary to monitor actively in high-risk groups such as health care staff. Hospitals in Shanghai have taken actions in preventive surveillance measures since 2020. The precision of determining the scope of screening and rapid contact tracing at the early stage for an outbreak has so far been successfully applied for COVID-19 infection control, and is believed to be cost-effective. Shanghai has so far declared four medium-risk areas. These areas are small in size with only 7.3, 5.1, 3.0 and 5.1 km^2^ respectively. Residents in medium-risk areas are advised not to leave Shanghai, unless providing a negative nucleic acid test result within 7 days. It is necessary to accurately designate risk areas to contain the spread of the epidemic, but also reduce the impact on the life of residents and the waste of resources.

Systematic and standardized prevention and control measures are as follows. First, a measure for prevention is active monitoring. Regular surveillance and testing of high-risk populations may identify infected people before widespread transmission occurs [4]. Second, it is superior to conduct precise epidemiological investigation together with medical observation and nucleic acid testing. It is of great importance for high-quality epidemiological investigation, timely contact tracing and quarantine of high-risk individuals.

## Conclusions

Active monitoring, precise epidemiological investigation and timely nucleic acid testing help to discover new cases, minimize the scope of screening, and interrupt the transmission. Mass-quarantine and mass nucleic acid testing could derail the economic vitality. It requires more precise and detailed strategies in the future, especially in metropolis.

Data were collected from the Shanghai Municipal Health Commission website (http://wsjkw.sh.gov.cn/) from January 21 to 24, 2021, including the number of daily new confirmed cases, the relationships among confirmed patients and the corresponding prevention and control measures. This article utilized government’s open data for analysis.

## Data Availability

The dataset analysed during the current study are available from the Shanghai Municipal Health Commission website (http://wsjkw.sh.gov.cn/).

## Abbreviations

SARS-CoV-2: Severe Acute Respiratory Syndrome Coronavirus 2
COVID-19: Coronavirus Disease 2019
